# Solvent and Flow Rate Effects on the Observed Compositional Profiles and the Relative Intensities of Radical and Protonated Species in Atmospheric Pressure Photoionization Mass Spectrometry

**DOI:** 10.1021/acs.analchem.1c03463

**Published:** 2022-03-14

**Authors:** Mary J. Thomas, Ho Yi Holly Chan, Diana Catalina Palacio Lozano, Mark P. Barrow

**Affiliations:** † Molecular Analytical Sciences Centre for Doctoral Training, University of Warwick, Coventry CV4 7AL, England; ‡ Department of Chemistry, University of Warwick, Coventry CV4 7AL, England

## Abstract

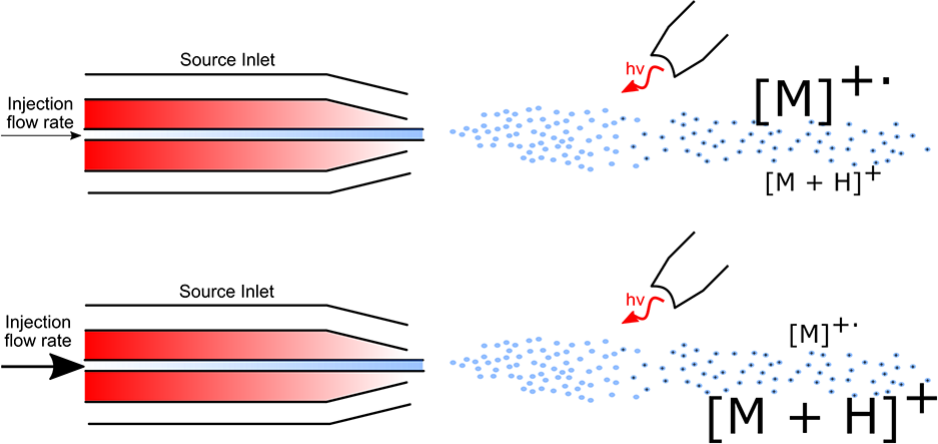

Sample preparation and instrument parameters have regularly been demonstrated to impact upon the observed results in atmospheric pressure photoionization, mass spectrometry (MS), and analytical techniques in general but may be overlooked when such methods are applied to the characterization of real-world samples. An initial investigation into different solvent systems demonstrated that the inclusion of ethyl acetate inverted the ratio of relative intensities of radical and protonated species (R/P). Design of experiments was performed and indicated that the injection flow rate is also a significant factor. The impact of the solvent system and flow rate on signal intensity, the observed compositional profile, and R/P of selected molecular groups is demonstrated further. An inversion of R/P is observed at higher flow rates in solvent systems commonly used in petroleomics studies, effecting a loss of molecular speciation. The findings presented reiterate the critical importance in considering experimental parameters when interpreting the results of analytical procedures.

Ultra-high resolution mass spectrometry (MS) techniques, such as Fourier transform ion cyclotron resonance (FTICR) and Orbitrap MS, offer unrivaled performance for the analysis of complex mixtures including petroleum.^1,2^ Atmospheric pressure photoionization (APPI) is an ionization technique regularly used for petroleum analysis, providing a broad overview of molecular composition by accessing both polar and non-polar compounds.^3^ Sample preparation procedures and instrument operating conditions have been shown to greatly affect the species and compositional profile observed in studies employing a range of ionization techniques, including electrospray ionization (ESI), atmospheric pressure laser ionization (APLI), atmospheric pressure chemical ionization (APCI), laser desorption ionization (LDI), laser-induced acoustic desorption (LIAD), and direct analysis in real time (DART).^4−14^ Furthermore, while some petroleomics researchers have observed and predicted differing results in APPI experiments^15−17^ by varying factors well known to affect the relative intensities of molecular species and ion-types ratios,^18^ often, these are not carefully considered prior to the analysis of complex samples using this ionization technique. To systematically explore these effects, crude oil is used an example complex mixture in this study.

Toluene is one of the most widely used solvents in studies employing APPI MS,^19^ sometimes in combination with other solvents such as propan-2-ol,^20^ owing to the dopant effect toluene afforded during gas-phase ionization.^21^ Several reaction pathways are known to influence the relative intensity ratio of radical ions and protonated species (R/P) observed in APPI experiments. For instance, dopant ions may react with solvent molecules and in turn by proton transfer with analytes possessing a high proton affinity (PA).^18^ PAHs dissolved in toluene have been shown to preferentially form protonated species at high vaporizer temperatures,^22^ with protonation likely proceeding via a two-step electron transfer and hydrogen transfer mechanism.^23^ The mechanism for dopant-assisted APPI is shown in eq 1.^24^
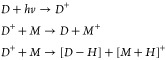
1

Species that prefer to ionize via protonation pathways may not always be detected when toluene is used solely as a solvent system for complex mixture analysis. For example, the N[H] class is well-known to comprise a large proportion of an observed petroleum profile by ESI. The N[H] class may also be observed using APPI and is typically representative of pyridinic compounds, in contrast to the N class, which typically corresponds to pyrrolic compounds,^25^ and the use of a more protic solvent, such as propan-2-ol, in combination with toluene can provide easier access to this class and to a wider range of molecular classes overall.^20^ The presence of protic solvents has also been demonstrated to significantly increase the [M + H]^+^ yield of PAHs in headspace APPI analyses.^23^ Other analyses of petroleum-related mixtures have employed solvent systems comprising dichloromethane (DCM)^26^ or acetonitrile (ACN).^27^

With conventional resources diminishing, oil production is shifting to heavier reserves, such as the oil sands bitumen mined in the Athabasca region of Alberta, Canada. Approximately three barrels of water are required to produce one barrel of synthetic crude oil,^5^ generating large volumes of oil sands process-affected water (OSPW). OSPW is a complex mixture that cannot be discharged into the environment, and so is stored in expansive tailings ponds.^28^ It was recently suggested that extraction of petroleum-related organic components can be maximized utilizing solutions predominantly comprising ethyl acetate (EA), rather than the more routinely used DCM.^6^ Improved access to the compositional space may be afforded,^29,30^ and the resulting profiles have been considered useful for statistical comparison to environmental samples.^31^ To the best of our knowledge, the effect of these solvents on the observed profile has not yet been investigated with respect to the analysis of crude oil.

APPI offers a large dynamic range and superior sensitivity at a low flow rate, particularly when compared to APCI, a related ionization method that leads to similar ion chemistries.^24,32^ Among the advantages over ESI, which tends to suffer greater ion suppression effects, is the possibility of quantitative analyses based upon knowledge of the ionization cross section. However, higher flow rates have been found to cause a decrease in signal intensity in APPI experiments, possibly due to larger ionization volumes limiting the distance traveled by light emitted from a krypton lamp, resulting in photon absorption by solvent vapor. A loss of dopant radical cations was suspected, leading to a decrease in response from analytes forming ions through charge exchange, and while analytes proceeding via proton transfer pathways were not as seriously affected, some saturation of their signal was observed at higher flow rates.^33^ Other studies have also suggested that light penetration through the sample volume is limited at higher flow rates, reducing the efficiency of ionization and that chemical ionization or photoionization followed by hydrogen abstraction can lead to increased formation of [M + H]^+^ ions under a variety of APPI conditions.^34^ Although analytes are routinely introduced along with a solvent system for direct infusion analyses, solvent-free evolved gas analysis techniques and those utilizing gas chromatography (GC) introduction with APPI and APLI can eliminate concerns over solvent effects for certain applications.^8,35^

Flow rates ranging from 180 to 3000 μL h^–1^ are typically used in petroleomics,^20,26,36−46^ and differences in the preferential formation of protonated species^36^ or radical ions^41,42^ has been observed. A systematic assessment of the effect of flow rate when applying (+) APPI-FTICR to the study of petroleum-related samples has not been carried out, and so the impact of varying this parameter upon the results obtained is not known. Although the radical and protonated classes can be used to differentiate pyrrolic and pyridinic nitrogen-containing classes^25^ and thiophenic and sulfidic sulfur-containing classes,^47^ respectively, the work presented here demonstrates that a loss of speciation may occur when higher flow rates are used. Increased deuterated ion formation has been observed at higher flow rates of both analyte and deuterated methanol solutions,^17^ with deuterated pyrrolic species also formed preferentially, in hydrogen–deuterium exchange studies. Such a loss of speciation may lead to amalgamation of protonated and radical classes, and in turn cause researchers to report only the double bond equivalents (DBE) value for the neutral molecule.

Design of experiments (DoE) has previously been used to optimize experimental parameters for ESI^48^ and APPI-FTICR MS^49^ analysis of petroleum samples, with an flow rate of 3000 μL h^–1^ that is suggested to be optimal for positive-mode (+) APPI analysis of crude oils.^50^ However, the responses considered were factors such as the maximum intensity, total number of ions, or molecular classes generated; the R/P was not reported. In this work, DoE was carried out with R/P as the response factor to improve understanding of the impact of the solvent system, sample concentration, and flow rate on the observed (+) APPI profile. The investigation into solvent effects and flow rate and the DoE study were carried out in parallel, with injection flow rates ranging between 400 and 4000 μL h^–1^ investigated.

## Experimental Section

### Sample Preparation

An Iraqi crude oil (ONTA, Toronto, Ontario, Canada) was dissolved at 0.05 mg mL^–1^ in toluene (Honeywell Speciality Chemicals Seelze GmbH, Hanover, Germany) and mixtures comprising toluene and propan-2-ol (Honeywell Speciality Chemicals Seelze GmbH, Hanover, Germany), chloroform (Merck KGaA, Darmstadt, Hesse, Germany), ACN, DCM, EA, *n*-hexane, chloroform, acetone, ethanol (Fisher Scientific, Hemel Hempstead, Hertfordshire, U.K.), and acetic acid (Fluka Analytical, Munich, Bavaria, Germany). A 1 mg mL^–1^ solution of 1,2-benzodiphenylene sulfide (Sigma-Aldrich Company Ltd., Gillingham, Dorset, United Kingdom) in toluene stock solution was spiked at 1% into 0.05 mg mL^–1^ solutions of the Iraqi crude oil for experiments involving a model compound. A South American crude oil was dissolved at concentrations of 0.05, 0.175, and 0.300 mg mL^–1^ in mixtures of toluene and propan-2-ol for the DoE study. Polarity indices, solvent groups,^51^ pKa values, vapor pressures, boiling points, proton affinities, and ionization efficiencies^52−54^ of the solvents used in this study are shown in Table S1.

### APPI-FTICR MS

Mass spectra were acquired using a 12T solariX Fourier transform ion cyclotron resonance (FTICR) mass spectrometer (Bruker Daltonik GmbH, Bremen, Germany), coupled to an APPI II source operated in positive-ion (+) mode. Nitrogen was used as the drying gas at a temperature of 220 °C and at an flow rate of 4 L min^–1^. The nebulizing gas was nitrogen and was maintained at a pressure of 1.2 bar. A krypton lamp was used to produce photons with energies of 10.0 and 10.6 eV. Samples were introduced by direct infusion using a syringe pump at a rate of 800 μL h^–1^ for the solvent study, ranging from 400–3600 μL h^–1^ for the flow rate study, and at rates of 600, 2300, and 4000 μL h^–1^ for the DoE study, without the activation of in-source dissociation.

4M data sets were acquired in the detection range *m*/*z* 147–1800 for 400 scans for the solvent study or 100 scans for the flow rate study; for the DoE study, the detection range *m*/*z* 221–1500 was used and 50 scans were acquired. The DoE was a 2^3^ full factorial design with five center points. Further details of the DoE variables are found in Table S2.

### Data Processing

The data were zero-filled once and apodized using a Sine-Bell function prior to applying a fast Fourier transform. The solvent study and DoE spectra were phased with a Half Hanning apodization (Kilgour) setting of 0.2–0.6 before baseline correction using FTMS Processing 2.1.0 (Bruker Scientific LLC, Billerica, MA, USA). Spectra were internally calibrated using homologous series and analyzed using DataAnalysis 4.2 (Bruker Daltonik GmbH, Bremen, Germany), prior to the data being imported into Composer 1.5.7 (Sierra Analytics, Modesto, CA, USA) for compositional analysis, with elemental constraints C = 0–200; H = 0–1000 ; N = 0–4; O = 0–4; S = 0–6 (Table S3). Aabel NG2 v.5.2 (Gigawiz Ltd. Co., Tulsa, Oklahoma, USA), and Origin 2016 (OriginLab Corporation, Northampton, MA, USA), was used for data visualization. Minitab Express 1.1.0 (Minitab LLC, State College, PA, USA) was used for DoE analysis.

## Results and Discussion

### Effect of Solvent on Observed Profile and Ion-Type Ratio

**Figure 1:**
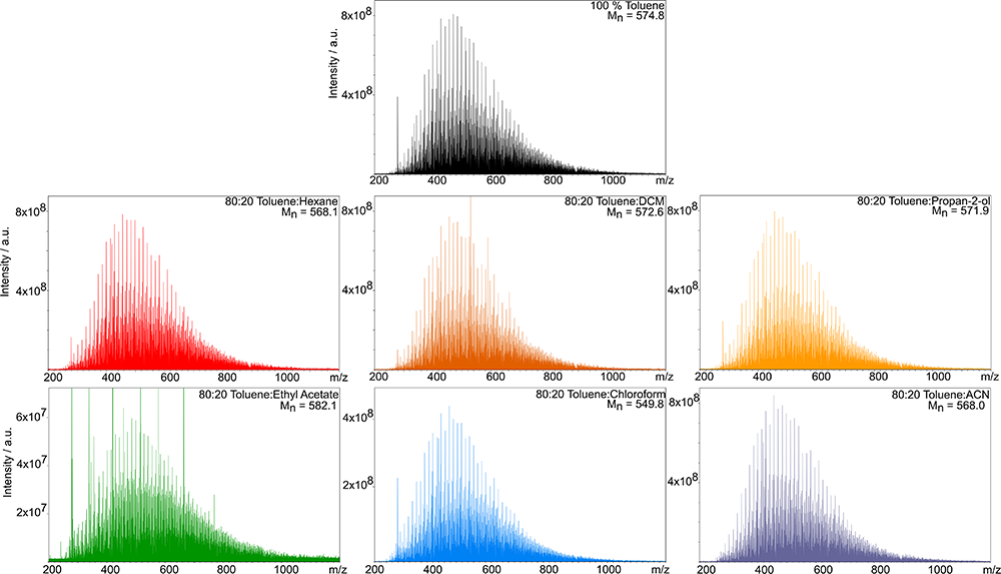
Enlarged (+) APPI mass spectra, showing crude oil distribution and *M_n_* in seven solvent systems.

Figure 1 shows the absorption mode (+) APPI mass spectra of the Iraqi crude oil in seven solvent systems, all investigated on the same day under the same instrument conditions. Although most mass envelopes and crude oil distributions are similar, the 80:20 toluene:EA is clearly different. As well as an approximately a factor of 10 decrease in signal intensity, a shift to higher *m*/*z* is observed with an increase in average molecular weight (*M_n_*) of 14.6.

**Figure 2:**
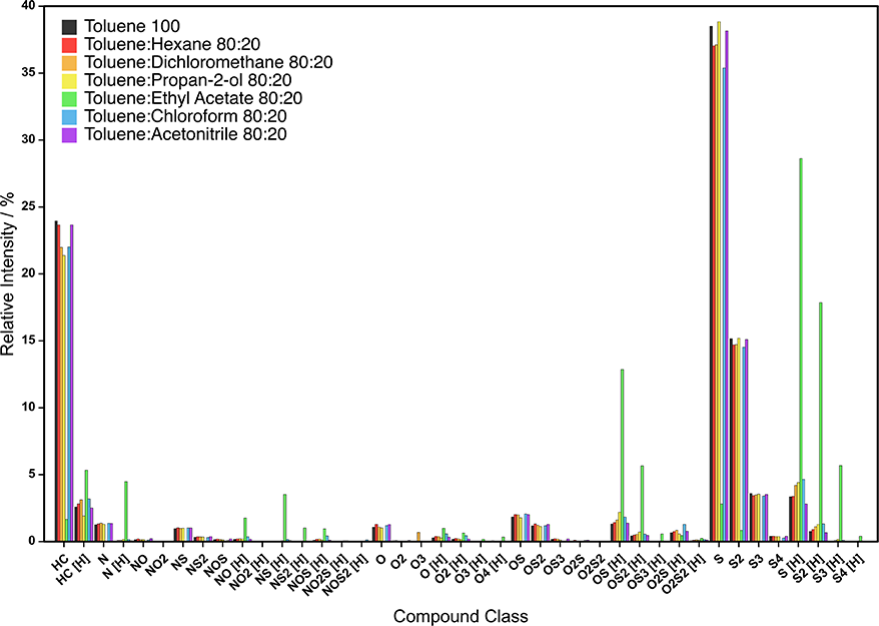
Effect of solvent system on compound class distribution.

The full compound class distribution for the Iraqi crude oil (Figure 2) demonstrates that the radical classes typically dominate, while the protonated classes made relatively low contributions to spectral intensity. The inverse was observed for the 80:20 toluene:EA solvent system. For instance, the average R/P over all the other the solvent systems is 7.26, in contrast to a ratio of 0.06 in 80:20 toluene:EA (Figure S1). The effect of the ethyl acetate cosolvent is particularly notable when comparing the R/P of species containing one or more sulfur atoms (Figure S2). The R/P of S-containing species has previously been used as an indicator of the most suitable experimental parameters in (+) APPI-FTICR MS analysis.^46^

Resonance stabilization of the protonated form of EA (Figure S3) may contribute to its high PA (Table S1). Furthermore, when generated in the manner shown, acetic acid may provide a source of labile protons, acting as a stronger acid in the gas-phase than HO_2_^•^.^55^ Interactions between ethyl acetate molecules may also increase the ionization volume, limiting analyte photo-absorption, causing both a loss of signal intensity and predominance of [M + H]^+^ ions.^33^

**Figure 3:**
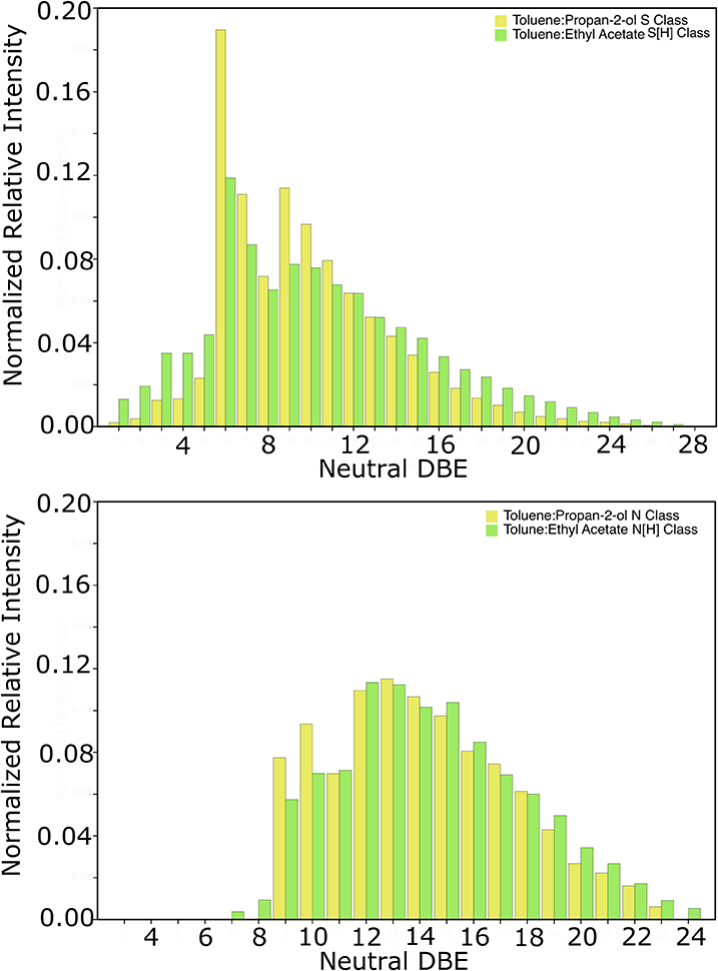
DBE distributions demonstrating the similarity between the radical S and N and protonated S[H] and N[H] classes in toluene:propan-2-ol and toluene:EA solvent systems.

The DBE range of the N[H] and S[H] classes in the toluene:EA solvent system is similar to those typically observed for the N and S classes, suggesting a loss of molecular speciation by ion-type. This is exemplified in Figure 3 and explored further in the DBE plots and distributions shown in Figures S4–S7.

The findings in Figure 3 and Figure S4 suggest that both pyridines and carbazoles ionize via protonation pathways in the toluene:EA solvent system, causing a loss of speciation between N and N[H] classes. Although the number of assignments made to the N[H] class is low for the toluene:propan-2-ol solvent system, the first intense homologous series is at DBE 8.5, while there is also a relatively intense homologous series of DBE 11.5 (Figures S4 and S8). This separation of 3 DBE is characteristic of pyridinic species.^25^ In the toluene:EA solvent system, however, this pattern is lost and the N[H] class is densely populated. Hydrogen–deuterium exchange studies^17, 57,^ or hyphenated chromatography FTICR MS experiments^26^ in which the extracted ion chromatogram (EIC) traces of both ion-type molecular assignments were compared could be used to confirm whether radical molecules in a given solvent system were instead proceeding via protonation pathways in another.

### Further Solvent Systems and Correlating Cosolvent Properties with the Observed Ion-Type Ratio

To investigate the factors underlying the observations made initially for the toluene:EA solvent system, the Iraqi crude oil was studied in four additional solvent systems:

(i) 80:20 toluene:acetone, as acetone also has a low ionization efficiency and high PA,
(ii) 99:1 toluene:EA, for a lower EA volume fraction,
(iii) 79.75:19.75 toluene:ethanol, with acetic acid added at 0.5% to determine whether the effect is due to hydrolysis of EA (Figure S3), and
(iv) 79.75:19.75 toluene:propan-2-ol, with acetic acid added at 0.5% to assess whether it altered the R/P of the 80:20 toluene:propan-2-ol system.

**Figure 4:**
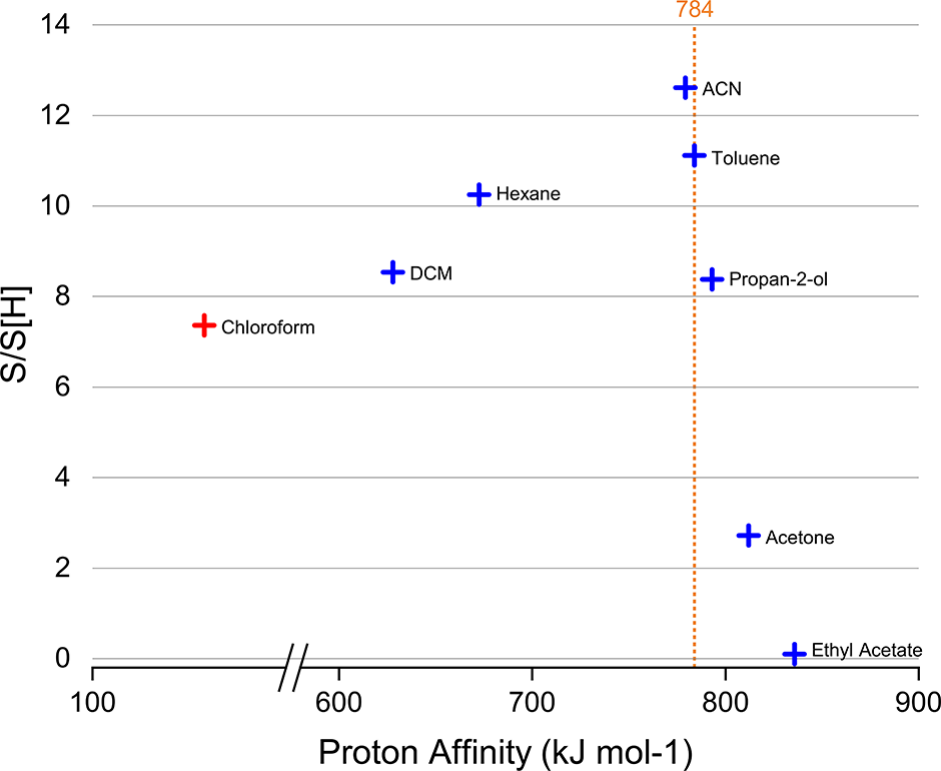
Change in S/S[H] ratio with PA of the 20 v/v % solvent. The PA of chloroform (red) is a calculated value, while the PAs of common solvents (blue) are experimentally determined.

Figure 2 shows that the S[H] class (protonated species) and S class (radical ions) contributed the greatest spectral intensity in the toluene:EA and other initial solvent systems. The ratio of relative intensities of these compound classes was therefore monitored as an indicator of protonated species formation, particularly as benzothiophenic species are usually detected in the radical S class^47^ starting at a DBE of 6. The normalized relative intensities of the S and S[H] classes in these further solvent systems are compared against those originally investigated in Figure S9. The findings shown suggest that hydrolysis of ethyl acetate can be ruled out as the mechanism underlying the preferential formation of protonated species, as the normalized relative intensity of S and S[H] classes in the 79.75:19.75 toluene:ethanol with the 0.5% acetic acid solvent system closely reflect that of the 80:20 toluene:propan-2-ol solvent system. With a volume fraction of just 1% in the solvent system, however, EA still effects a reduction in the ion-type ratio, although the effect is less profound than when EA is at 20%. Factors considered to have a possible impact on R/P include solvent volatility, ionization energy, and PA. Figures S10–S12 show no correlation, however, between the R/P and the total vapor pressure of the solvent system (calculated using Raoult’s Law) or the volatility or ionization energy of the 20% v/v solvent. In APPI, charge exchange is widely considered to be favored by lower PA solvents, while proton transfer is favored by higher PA solvents;^57^ however, Figure 4 suggests that the S/S[H] ratio increases with cosolvent PA up to ca. 800 kJ mol^–1^, above which the ratio decreases. This trend is established tentatively, given the lack of viable solvents to extend the range of data points; examples of molecular entities with PA between 157.8 and 628 kJ mol^–1^ are given in Table S4. A previous study into the effect of solvent on ESI spectra covered a range of chlorinated variants of methane and ethane;^58^ however, no PA could be established (or only calculated) for these molecular entities. The lower R/P observed in the 80:20 toluene:acetone solvent system may be due to the relatively high PA of acetone.

Figure 4 suggests that the PA of the solvent system must be carefully considered as it can influence the R/P and APPI profile. This is especially important when comparing results between studies where different sample preparation steps or hyphenation steps are employed, for example, where high PA solvents are used alone or in combination with other solvents in liquid chromatography (LC) or for extraction of analyte compounds of interest. Other ionization methods, particularly ESI, are known to suffer ionization suppression,^59,60^ particularly when analytes are in the presence of large, more highly charged molecules, salts, or other non-volatile solute or when the pH of the solvent system is varied.^6,61^ Therefore, the impact of solvent choice on ionization has implications beyond petroleomics and APPI experiments, particularly in emerging fields such as quantitative proteomics, where LC is widely used with a range of solvent mobile phases.^62,63^

### Design of Experiment (DoE) Study with Ion-Type Ratio as Response Factor

A basic DoE study was carried out to indicate whether concentration, toluene solvent fraction, or flow rate had significant effect on the R/P observed (magnitude and direction shown in Figure S13 and standardized effects shown in Figure S14). Because flow rate is varied widely in the field while the impact upon the results obtained remains poorly understood, this was among the experimental parameters targeted. All factors, and most of their interactions, were found to be significant, while non-linear effects is suggested given that the R/P at the center point does not sit on a straight line between the minimum and maximum values. The toluene volume fraction and the flow rate were found to be the main factors influencing R/P, with a less significant effect demonstrated for the sample concentration and its interactions with the other factors. Samples prepared with 100% toluene volume fraction effected the highest R/P response, while the highest flow rate gave a lower response.

**Figure 5:**
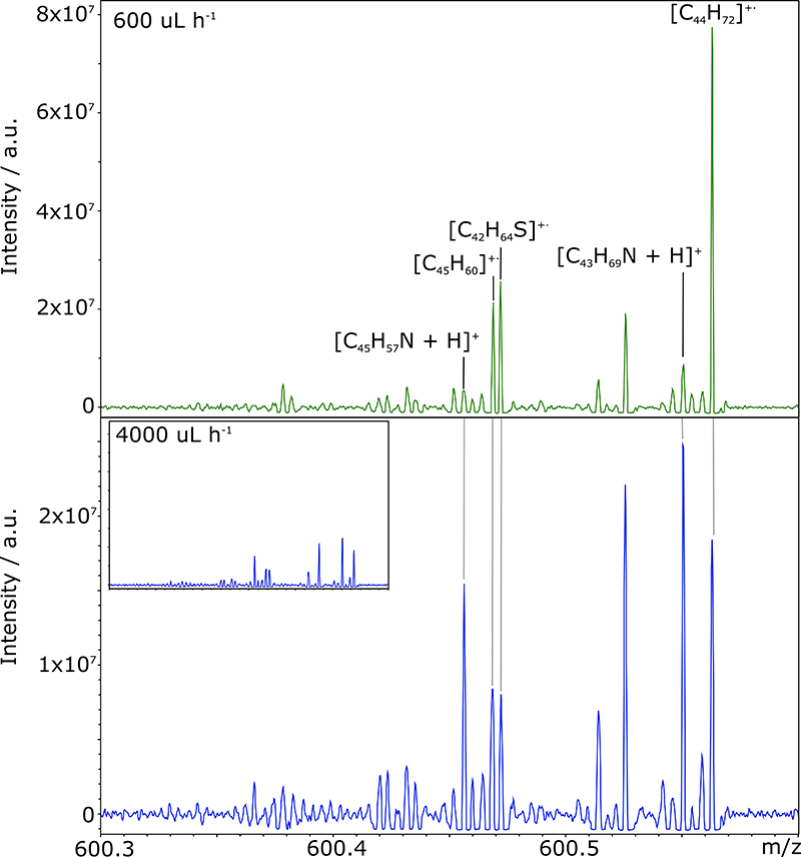
Enlarged phase corrected MS regions from DoE experiments and flow rates of 600 and 4000 μL h^–1^. A decrease in overall intensity (inset on intensity scale equivalent to upper panel), a shift in ion-type predominance, and higher response for species assigned to the N[H] class are observed at higher flow rate.

To illustrate the effect observed when only the flow rate is altered and the concentration and toluene volume fraction are kept constant, enlarged regions of the DoE mass spectra of the South American crude oil at 600 and 4000 μL h^–1^ are shown in Figure 5. These demonstrate the decrease in intensity at high flow rate and the more severe effect on radical ions assigned to the HC and S classes. The intensity of peak assigned to the N[H] class has increased, however, and dominates the spectrum at 4000 μL h^–1^. Predominant N[H] peaks in the same spectral window have been observed in an APPI study of asphaltene fractions; however, the flow rate used was not reported.^64^ As flow rate was indicated by this simple DoE to have a significant effect, a more thorough investigation over a range of flow rates is discussed in the section that follows. A fuller DoE with prior calculation of a parameter matrix to account for the variability and range of experimental procedures analytical measurements would be a valuable, although time-consuming, alternative approach.

### Effect of Flow Rate on Ion-Type Ratio

**Figure 6:**
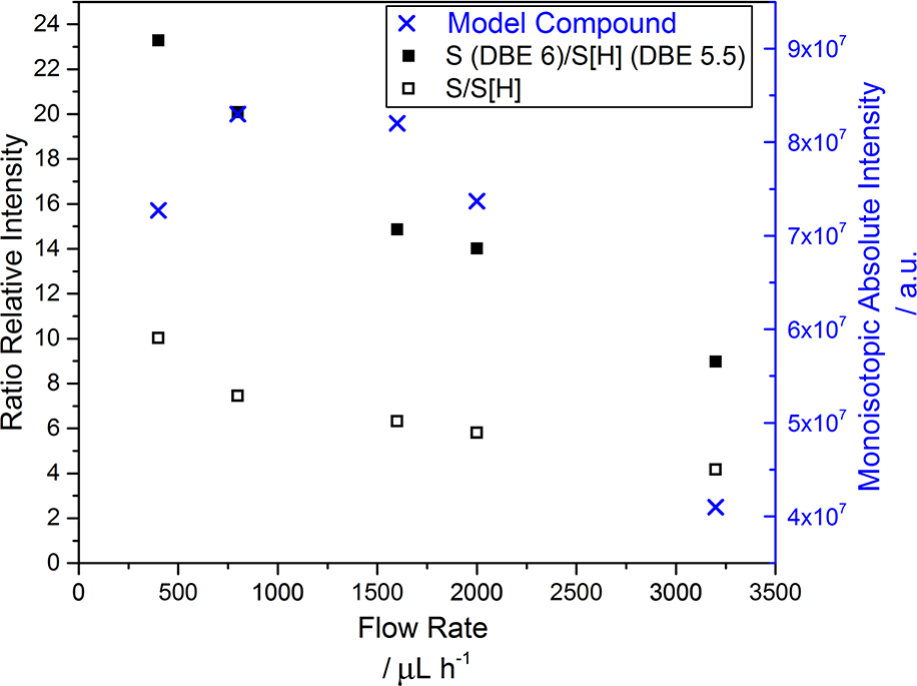
Change with increasing flow rate in: S/S[H], homologous series DBE 6/5.5, and model compound intensity.

The crude oil in the toluene:propan-2-ol solvent system was initially studied at flow rates ranging from 400–3200 μL h^–1^. In parallel, the same system was spiked with thiophenic compound 1,2-benzodiphenylene sulfide, a sulfur-containing species with a neutral DBE of 12. Consistent with other studies of model compounds by APPI,^56^ the protonated species was not observed, possibly due to a lack of alkylation about its core structure; however, the radical ion was detected. The monoisotopic absolute intensity of the radical ion in the spiked system is compared against the S/S[H] class and DBE 6/5.5 ratios detected in the crude oil system in Figure 6.

Figure 6 shows that although the monoisotopic absolute intensity of the model compound does increase slightly between 400 and 800 μL h^–1^, remaining constant up to 1600 μL h^–1^, it decreases with increasing flow rate thereafter. A similar general decrease with increasing flow rate is observed in the toluene and 80:20 toluene:acetone solvent systems (Figure S15). In the 99:1 toluene:EA solvent system, however, the intensity is low at 1000 μL h^–1^ and at all other flow rates studied, with no change greater than 0.1% relative to the intensity at 1000 μL h^–1^.

**Figure 7:**
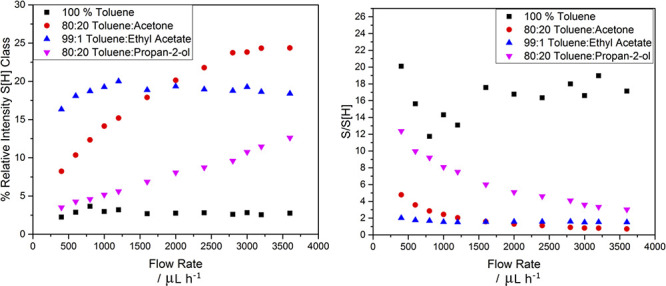
Change with increasing flow rate in S[H] class intensity and S/S[H] ratio with increasing flow rate in different solvent systems.

A non-linear decrease R/P, a result predicted by the DoE data, is also demonstrated in Figure 6. The change in relative abundance of the S[H] class and S/S[H] ratio in different solvent systems with varying flow rate is explored further in Figure 7. The relative intensity of the S[H] class shows a general increase with flow rate, except in toluene only, and in 99:1 toluene:ethyl acetate, where it appears to plateau above 1600 μL h^–1^. Similarly, the S/S[H] ratio decreases with increasing flow rate, particularly in 80:20 toluene:propan-2-ol and 80:20 toluene:acetone, the latter of which sees the ratio drop below 1, indicating the onset of the preferential formation and predominance of protonated species above 2800 μL h^–1^. Similar shifts in ion-type predominance from radical to protonated have been predicted in theoretical studies where the temperature of sample desolvation is increased.^15^

The results highlight the need for caution where higher flow rates are used in APPI, particularly for the study of complex mixtures. While a loss of signal intensity reduces sensitivity, such that lower abundance species may not be detected, the preferential formation of protonated species makes speciation of compound types less feasible, effecting a reduction in compositional access and loss of sample information. Furthermore, a species that is instead protonated at higher flow rate effectively has its fragmentation efficiency reduced, limiting the elucidation of structural information.

The factors underlying the decrease in radical ion intensity at high flow rates have been discussed elsewhere, with early APPI studies suggesting that dopant toluene ions must not be entirely consumed in dopant–solvent reactions for efficient radical ion formation. Higher flow rates are known to drive dopant–solvent reactions to completion.^65^ Other studies have shown that high flow rates produce larger solvent clusters, which drive recombination reactions, effectively lowering the sensitivity of APPI overall, with protonated species less severely affected.^16,33,66^

The impact of the solvent system and flow rate on the profile and R/P observed has implications for studies of real-world complex samples. Indeed, researchers working in the field of petroleomics have identified that flow rates exceeding 1500 μL h^–1^ have a negative impact on signal intensity,^67^ with formation of solvent clusters and their subsequent reaction with toluene dopant ions identified as a possible cause that more significantly impacts radical ion signal.^16^ Nevertheless, flow rate is often overlooked in petroleomics research and contrasting findings on observed ion-type ratios are therefore common. For example, in a recent study reporting a flow rate of 50 μL min^–1^ (i.e., 3000 μL h^–1^) many thousands of unique molecular formulae were assigned, the majority of which were protonated species, and it was subsequently inferred that APPI favors protonation of analyte components.^34^ In another study, reporting a record 244,779 assignments,^1^ APPI favored radical ions with an flow rate of 500 μL h^–1^. The differences in the ion-type ratio and preferential compound class detection reported across the literature are in line with the findings of this study.

## Conclusions

The solvent system and flow rate were found to have a significant impact on the signal intensity, crude oil profile, and ion-type ratio in (+) APPI-FTICR MS. The use of high PA solvents in combination with toluene and toluene volume fraction had the greatest impact. The use of high flow rates, widely utilized during petroleomics research without the impact being previously well understood, also inverts the ion-type ratio in several solvent systems.

The use of aprotic solvents with high PAs, such as EA and acetone, increased the number of protonated species detected, with species that would ordinarily ionize via radical pathways, such as thiophenes and pyrroles, appearing to ionize instead via protonation pathways. This affects a loss in molecular speciation, previously made possible through observing characteristic patterns in DBE plots and distributions. Although not as severe as the effect observed with EA as cosolvent, a trend of increasing protonated species formation and decreasing radical ion formation was observed in other solvent systems at high flow rate.

Experimental parameters, including the solvent system and flow rate, have a critical influence on the characterization of complex samples such as petroleum and are an important consideration when comparing compositional profiles. The widely reported observations of predominantly protonated species may be an ionization phenomenon, and care should be taken to determine whether experimental factors have influenced the results. The importance of solvent choice extends beyond petroleum analysis, particularly to hyphenated mass spectrometry and applications such as quantitative proteomics, metabolomics, and environmental science.

## Supporting Information Available

Solvent properties and experimental parameter details. Supplementary figures including additional DBE distributions and plots.
