# Supplementary material for: Solvent and Flow Rate Effects on the Observed Compositional Profiles and the Relative Intensities of Radical and Protonated Species in Atmospheric Pressure Photoionization Mass Spectrometry
Source: Anal Chem. 2022 Mar 14;94(12):4954–60. doi: 10.1021/acs.analchem.1c03463 (PMC8969439; doi:10.1021/acs.analchem.1c03463)
Supplement: Supplementary file 1 — ac1c03463_si_001.pdf [file ac1c03463_si_001.pdf]

Supporting Information

Solvent and Flow Rate Effects on the Observed Compositional Profiles and the Relative Intensities of Radical and Protonated Species in Atmospheric Pressure Photoionization Mass Spectrometry

Mary J. Thomas[1,2], Ho Yi Holly Chan[2], Diana Catalina Palacio Lozano[2], Mark P. Barrow[2*]

1. Molecular Analytical Sciences Centre for Doctoral Training, University of Warwick, Coventry, CV4 7AL
2. Department of Chemistry, University of Warwick, Coventry, CV4 7AL

*Corresponding Author M.P.Barrow@warwick.ac.uk

Table of Contents

Table S1. Properties of solvents used, with cosolvents used in the first and second stages of the study ordered by polarity index

Table S2. Design of Experiment Parameters. 3 factors (sample concentration, toluene fraction, and flow rate) were studied using a full factorial 2^3 design, using a single replicate of 13 runs including 5 centrepoints. All terms were free from aliasing

Table S3. Composer 1.5.7 parameters for the assignment of molecular formulae

Figure S1. Summed relative intensity of protonated and radical ion classes assigned in solvent systems initially studied

Figure S2. Summed relative intensity of protonated and radical ion classes containing at least one sulfur atom assigned in solvent systems initially studied

Figure S3. Hypothetical scheme illustrating ethyl acetate generating sources of labile protons; its resonance stabilized protonated form, and its hydrolysis product acetic acid. $[H]^+$ may be generated in the source as indicated in Equation scheme 1

Figure S4. DBE vs. carbon number plots for N and N[H] classes in toluene:propan-2-ol and toluene:ethyl acetate solvent systems, with squares surrounding corresponding DBE and carbon number values demonstrating the similarity between the species apparent in the N class and N[H] class of each, respectively. The N[H] class was observed at low relative intensity in the toluene:propan-2-ol solvent system, while the N class was not detected in the toluene:ethyl acetate solvent system

Figure S5. DBE vs. carbon number plots for S and S[H] classes in toluene:propan-2-ol and toluene:ethyl acetate solvent systems, demonstrating similarity between the species apparent in the S class and S[H] class of each, respectively. The S[H] class and S class were observed at low relative intensity in the toluene:propan-2-ol and toluene:ethyl acetate solvent systems respectively

Figure S6. Summed N and N[H] class DBE distribution for the toluene:propan-2-ol solvent system compared the N[H] class DBE distribution for the toluene:ethyl acetate solvent system

Figure S7. Summed S and S[H] class DBE distribution for the toluene:propan-2-ol solvent system compared the S[H] class DBE distribution for the toluene:ethyl acetate solvent system

Figure S8. DBE distribution of the N[H] class compared between the toluene:ethyl acetate and toluene:propan-2-ol solvent systems

Figure S9. Normalized relative intensities of S and S[H] classes for all solvent systems studied

Figure S10. Change in S/S[H] with total vapour pressure of solvent system

Figure S11. Change in S/S[H] with polarity index of solvent added at 20 v/v % to solvent system

Figure S12. Change in S/S[H] with ionization energy of solvent added at 20 v/v % to solvent system

Table S4. Molecular entities with proton affinities between 157.8 and 628 kJmol^{-1}. Due to the hazards associated with these, or their lack of stability and commercial availability, in liquid form, they are unsuitable to be used as cosolvents used in this study.

Figure S13. Summary of DoE study where response factor is the mean ratio of radical to protonated species (R/P). While higher sample concentration and larger toluene solvent fraction increase radical ion formation, a high flow rate decreases the relative proportion of radical species detected. Red squares are indicative of the effect at the centerpoint of the DoE.

Figure S14. Pareto chart of standardized effects for DoE experiments. The response is the mean ratio of radical and protonated species detected (R/P). Factors that exceed the dashed reference line are statistically significant at the $\alpha = 0.05$ level.

Figure S15. Percent change in monoisotopic absolute intensity of model compound spiked into Iraqi crude oil dissolved in different solvent systems with flow rate relative to observed intensity at 1000 μL h^{-1}.

Table S1. Properties of solvents used, with cosolvents used in the first and second stages of the study ordered by polarity index

Solvent	Polarity Index	pKa	Ionization Energy / eV	Vapor Pressure / kJ mol^{-1}	Boiling Point / K	Proton Affinity / kJ mol^{-1}
Toluene	2.3	41	8.828 ± 0.001	37 ± 3	383.8 ± 0.2	784.0
n-hexane	0.0	N/A	10.13 ± 0.10	31 ± 1	341.9 ± 0.3	672.5
DCM	3.4	N/A	11.33 ± 0.04	29.03 ± 0.08	313 ± 1	628.0
Propan-2-ol	4.3	17.1	10.17 ± 0.02	45 ± 3	355.5 ± 0.4	793.0
Ethyl acetate	4.3	25	10.01 ± 0.05	35 ± 2	350.2 ± 0.2	835.7
Chloroform	4.4	15.5	11.37 ± 0.02	31.32 ± 0.08	334.3 ± 0.2	157.8‡
Acetonitrile	6.2	25	12.20 ± 0.01	33.45 ± 0.21	354.8 ± 0.4	779.2
Acetone	5.1	19.6	9.703 ± 0.006	31.27	329.3 ± 0.3	812
Ethanol	5.2	16	10.48 ± 0.07	42.3 ± 0.4	351.5 ± 0.2	776.4
Acetic Acid	6.2	4.75	10.65 ± 0.02	51.6 ± 1.5	391.2 ± 0.6	783.7

‡Calculated value.

Table S2. Design of Experiment Parameters. 3 factors (sample concentration, toluene fraction, and flow rate) were studied using a full factorial 2^3 design, using a single replicate of 13 runs including 5 centrepoints. All terms were free from aliasing

Standard Order	Run Order	Centrepoint?	Blocks	Concentration / mg mL^{-1}	Toluene Fraction / %	Flow Rate / $\mu L\ h^{-1}$
2	1	No	1	0.300	20	600
4	2	No	1	0.300	100	600
10	3	Yes	1	0.175	60	2300
6	4	No	1	0.300	20	4000
3	5	No	1	0.050	100	600
11	6	Yes	1	0.175	60	2300
9	7	Yes	1	0.175	60	2300
8	8	No	1	0.300	100	4000
13	9	Yes	1	0.175	60	2300
7	10	No	1	0.050	100	4000
12	11	Yes	1	0.175	60	2300
1	12	No	1	0.050	20	600
5	13	No	1	0.050	20	4000

Table S3. Composer 1.5.7 parameters for the assignment of molecular formulae

**Parameter**	**Constraints**
Polarity	Positive
Ion properties	Adducts = H; allow radical and adduct/loss ions; remove isolated assignments
m/z range	m/z 200-1300
DBE range	-0.5 - 40
Element ranges	C = 0-200; H = 0-1000 ; N= 0-4; O = 0-4; S = 0-6

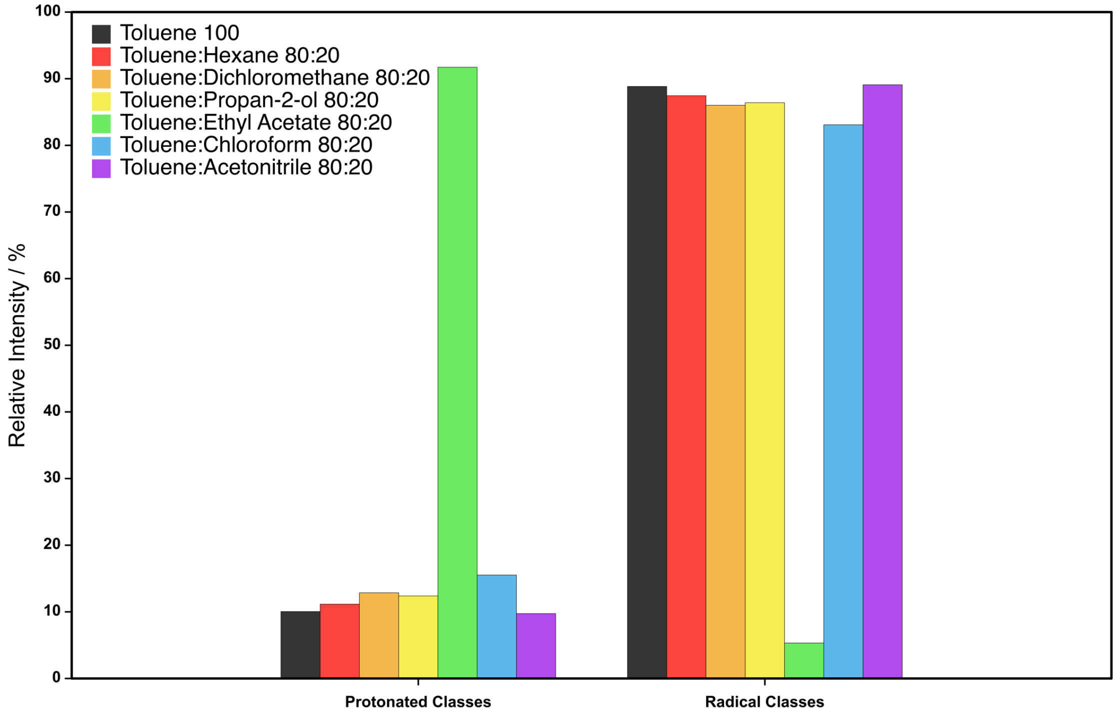

Figure S1. Summed relative intensity of protonated and radical ion classes assigned in solvent systems initially studied

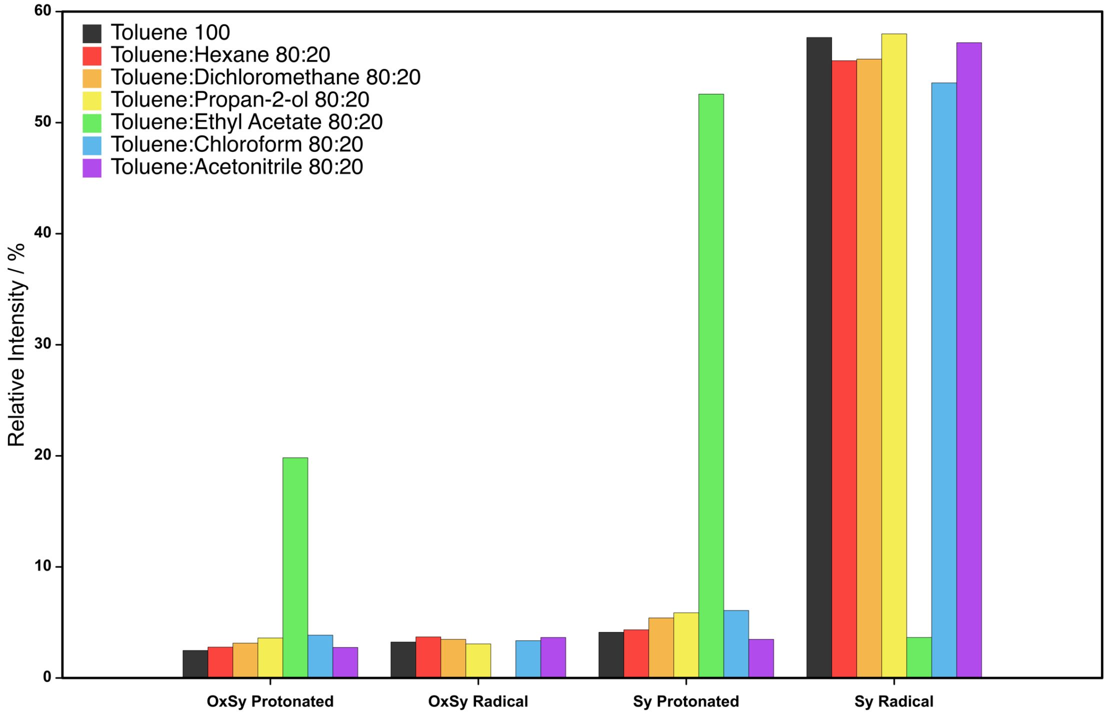

Figure S2. Summed relative intensity of protonated and radical ion classes containing at least one sulfur atom assigned in solvent systems initially studied

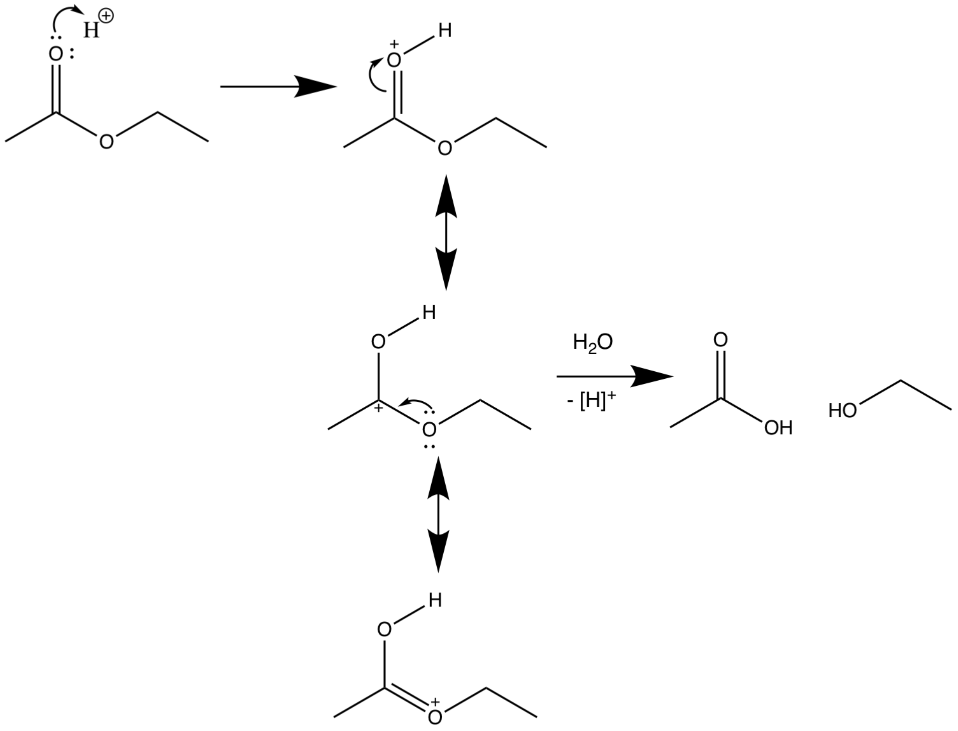

Figure S3. Hypothetical scheme illustrating ethyl acetate generating sources of labile protons; its resonance stabilized protonated form, and its hydrolysis product acetic acid. $[H]^+$ may be generated in the source as indicated in Equation scheme 1

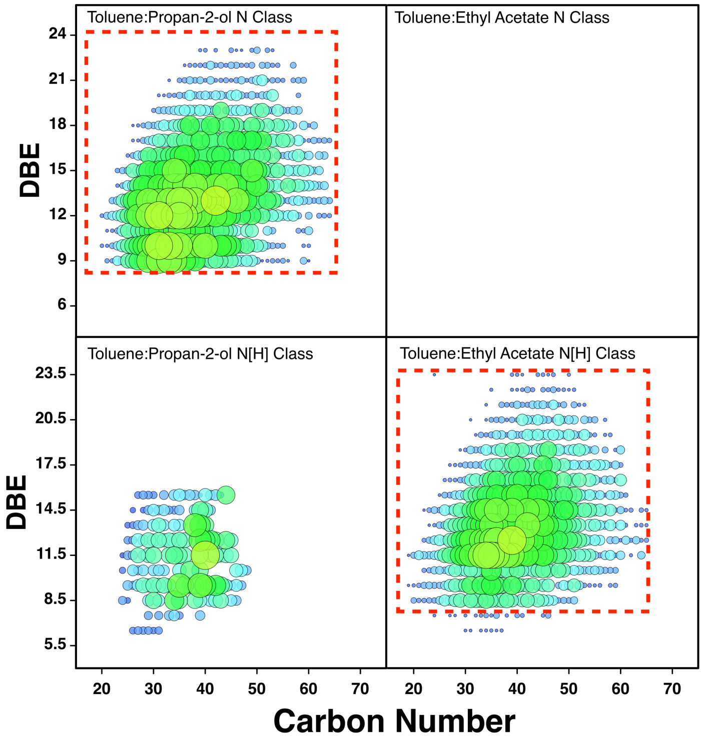

Figure S4. DBE vs. carbon number plots for N and N[H] classes in toluene:propan-2-ol and toluene:ethyl acetate solvent systems, with squares surrounding corresponding DBE and carbon number values demonstrating the similarity between the species apparent in the N class and N[H] class of each, respectively. The N[H] class was observed at low relative intensity in the toluene:propan-2-ol solvent system, while the N class was not detected in the toluene:ethyl acetate solvent system

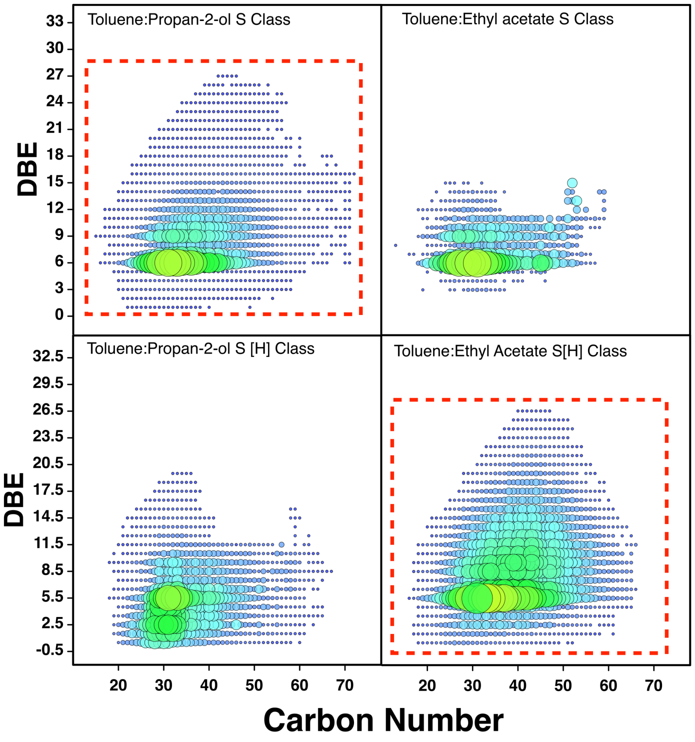

Figure S5. DBE vs. carbon number plots for S and S[H] classes in toluene:propan-2-ol and toluene:ethyl acetate solvent systems, demonstrating similarity between the species apparent in the S class and S[H] class of each, respectively. The S[H] class and S class were observed at low relative intensity in the toluene:propan-2-ol and toluene:ethyl acetate solvent systems respectively

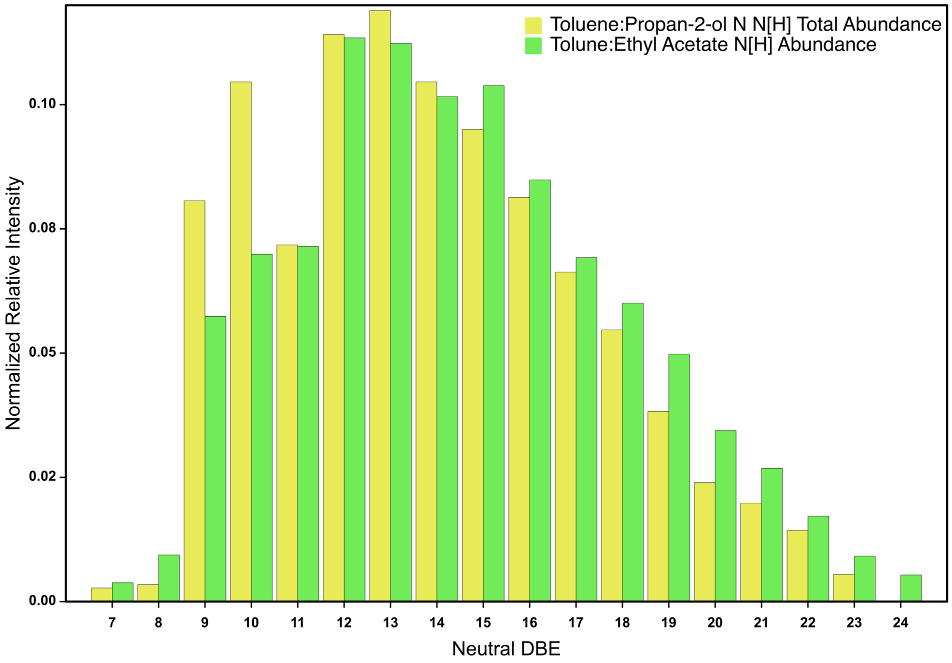

Figure S6. Summed N and N[H] class DBE distribution for the toluene:propan-2-ol solvent system compared the N[H] class DBE distribution for the toluene:ethyl acetate solvent system

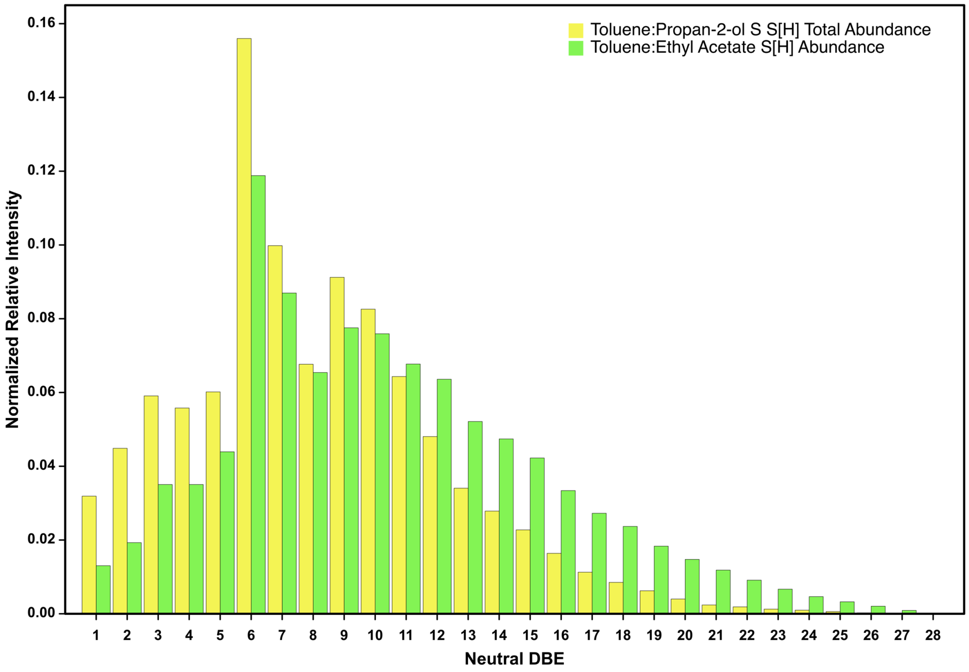

Figure S7. Summed S and S[H] class DBE distribution for the toluene:propan-2-ol solvent system compared the S[H] class DBE distribution for the toluene:ethyl acetate solvent system

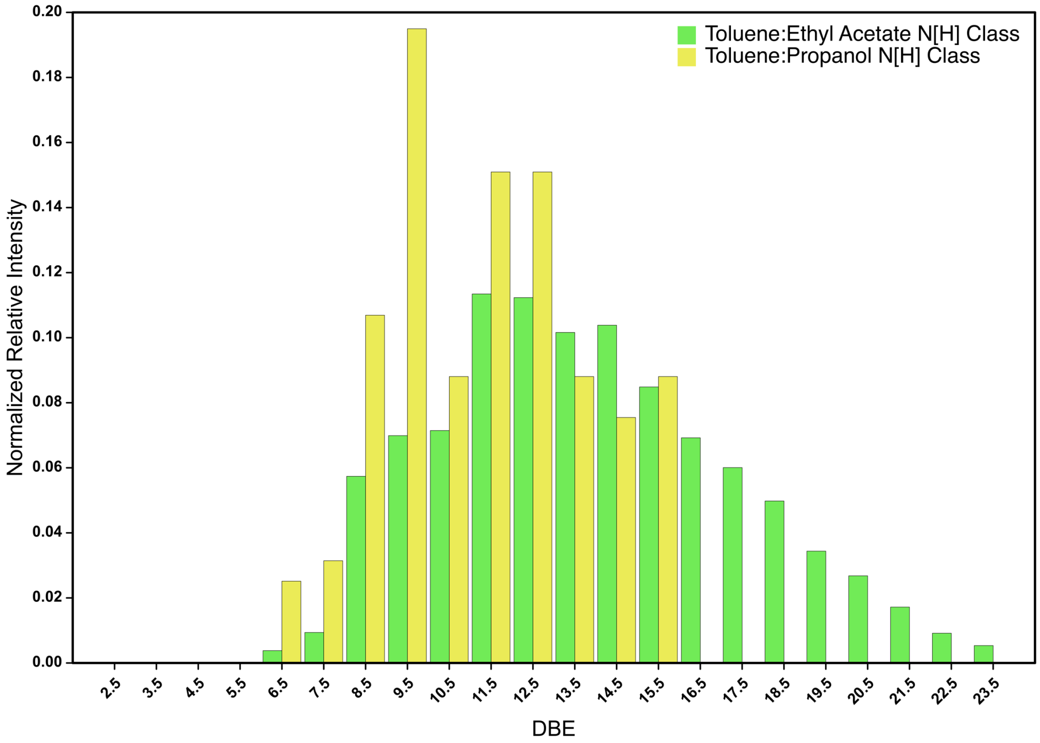

Figure S8. DBE distribution of the N[H] class compared between the toluene:ethyl acetate and toluene:propan-2-ol solvent systems

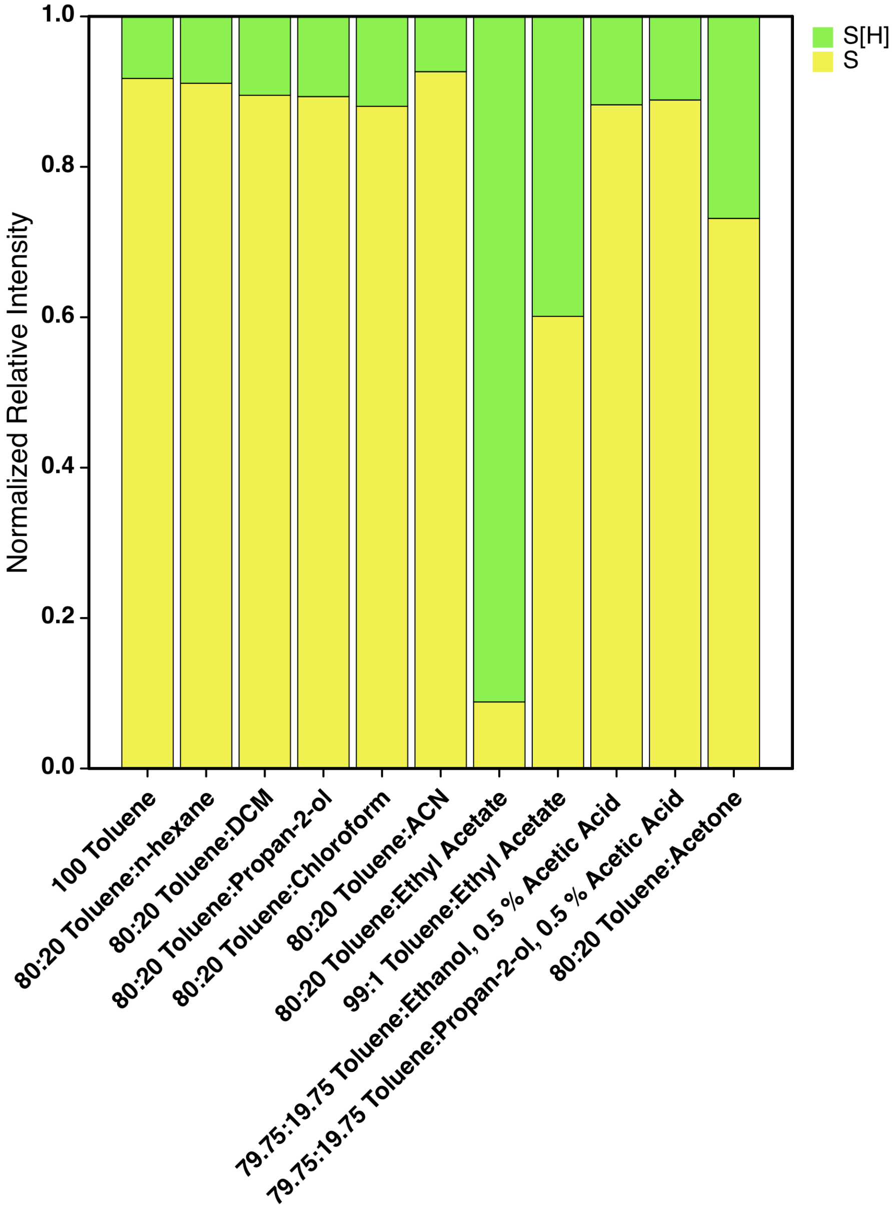

Figure S9. Normalized relative intensities of S and S[H] classes for all solvent systems studied

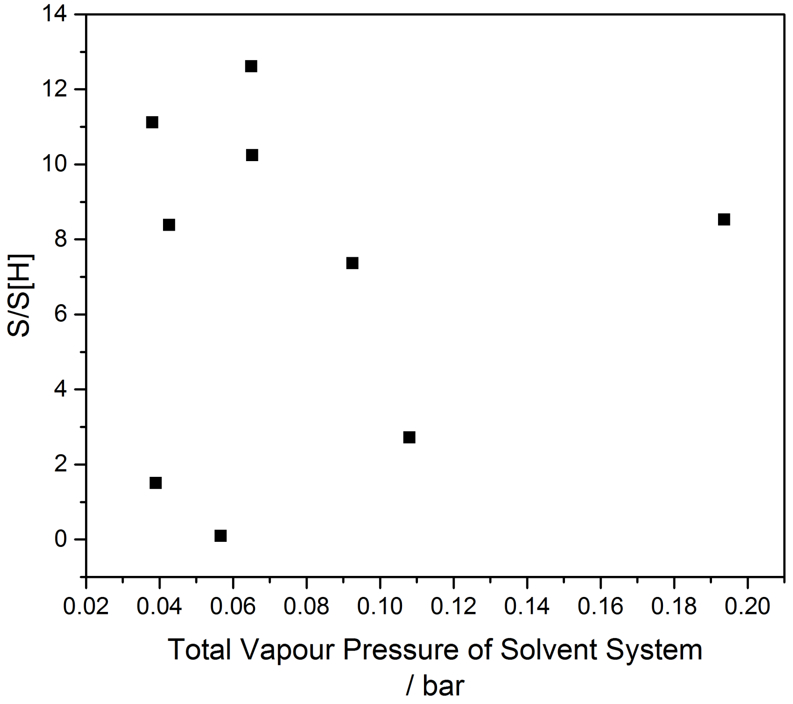

Figure S10. Change in S/S[H] with total vapour pressure of solvent system

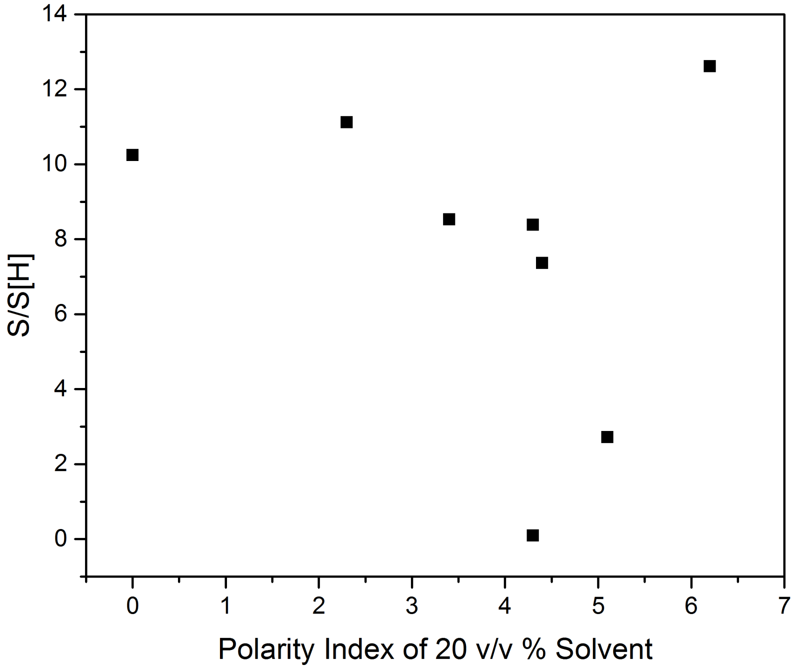

Figure S11. Change in S/S[H] with polarity index of solvent added at 20 v/v % to solvent system

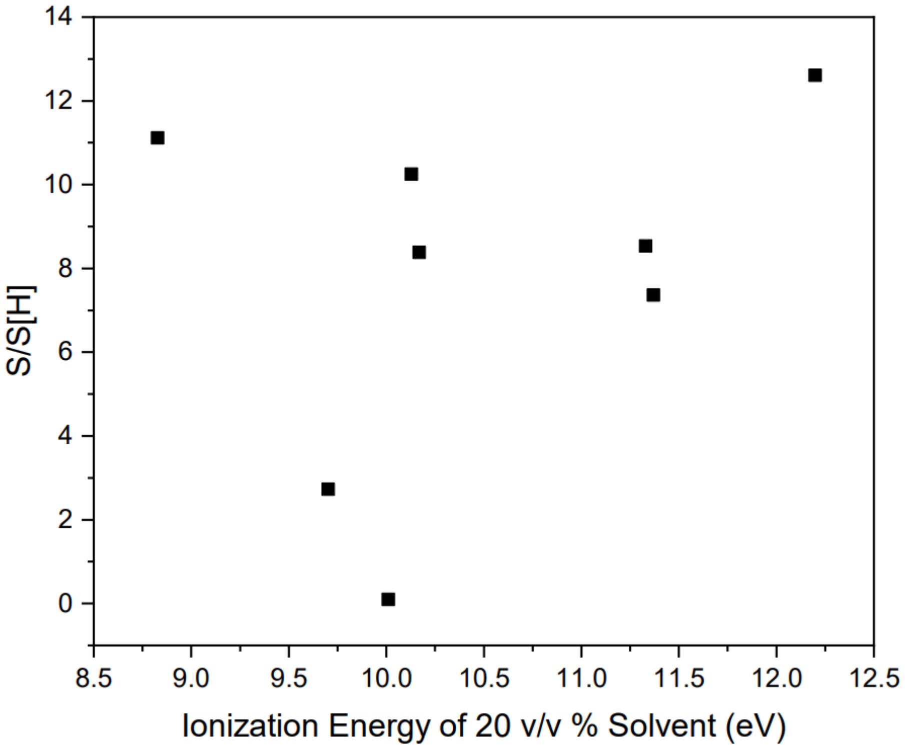

Figure S12. Change in S/S[H] with ionization energy of solvent added at 20 v/v % to solvent system

Table S4. Molecular entities with proton affinities between 157.8 and 628 kJmol^{-1}. Due to the hazards associated with these, or their lack of stability and commercial availability, in liquid form, they are unsuitable to be used as cosolvents used in this study.

**Molecular entity**	**Proton Affinity / kJ mol^{-1}**
Helium	177.8
Argon	369.2
Hydrogen fluoride	484.0
Nitric oxide	531.8
Carbon dioxide	540.5
Methane	543.5
Hydrogen chloride	556.9
Chlorotrifluoromethane	571.3
Ethane	596.3

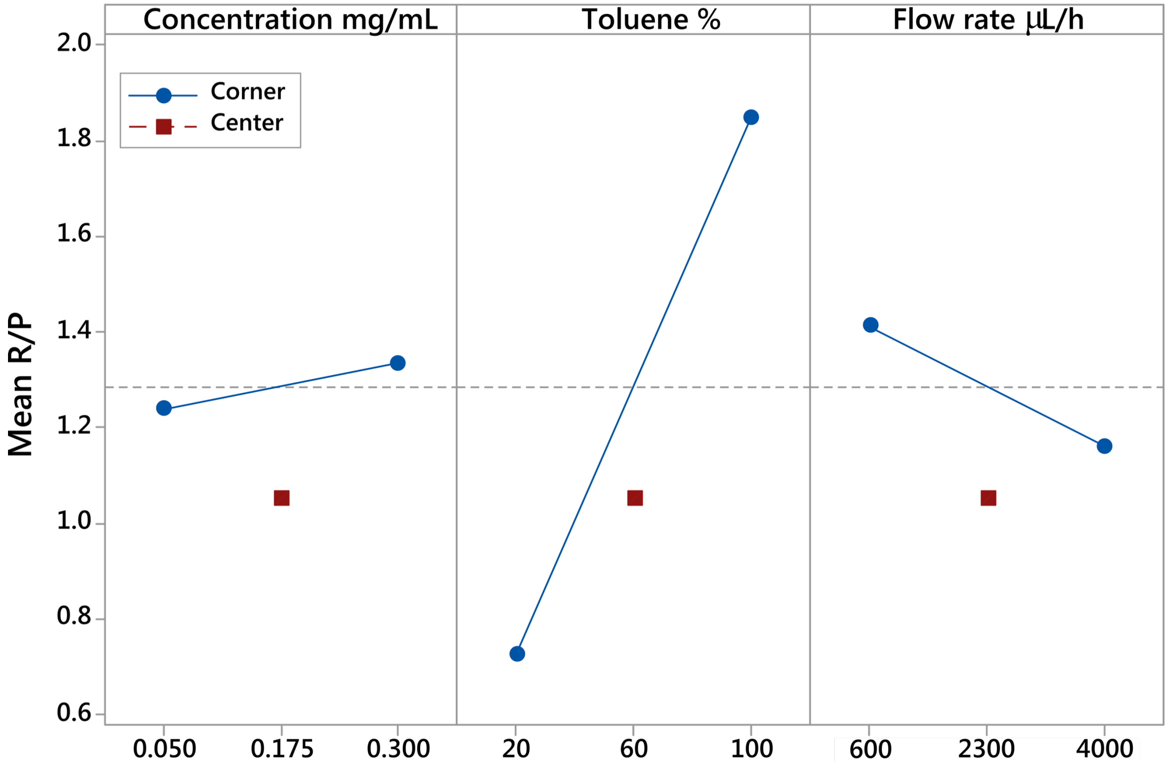

Figure S13. Summary of DoE study where response factor is the mean ratio of radical to protonated species (R/P). While higher sample concentration and larger toluene solvent fraction increase radical ion formation, a high flow rate decreases the relative proportion of radical species detected. Red squares are indicative of the effect at the centerpoint of the DoE.

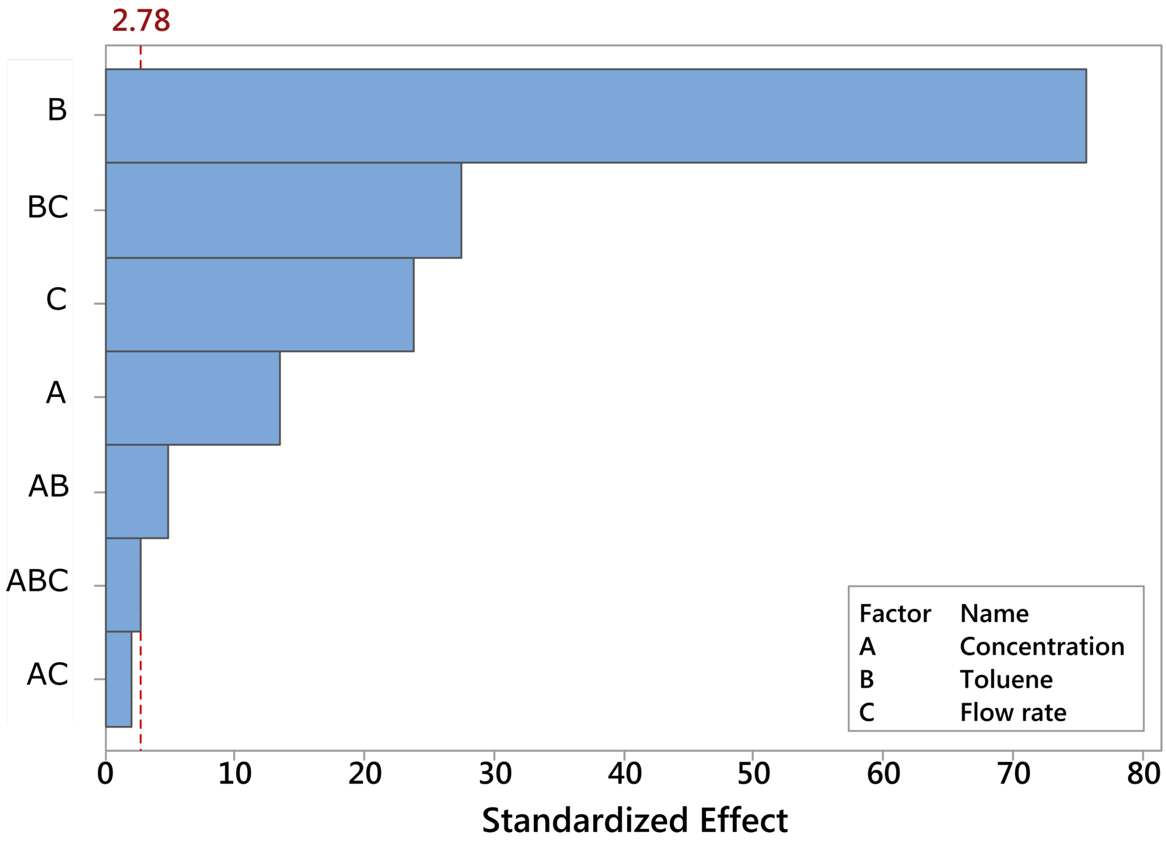

Figure S14. Pareto chart of standardized effects for DoE experiments. The response is the mean ratio of radical and protonated species detected (R/P). Factors that exceed the dashed reference line are statistically significant at the $\alpha = 0.05$ level.

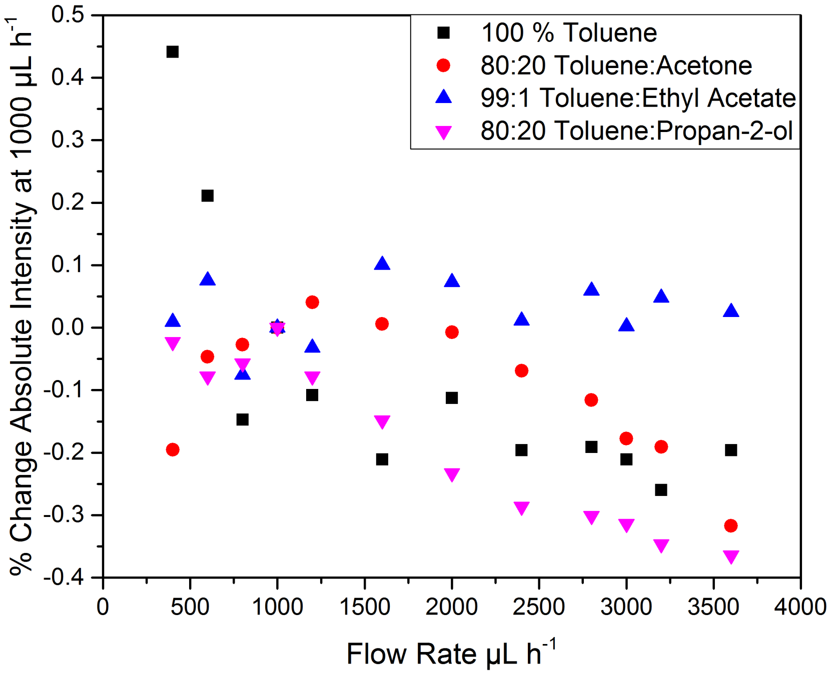

Figure S15. Percent change in monoisotopic absolute intensity of model compound spiked into Iraqi crude oil dissolved in different solvent systems with flow rate relative to observed intensity at 1000 μL h^{-1}.
